# Supplementary material for: Structure-forming CAG/CTG repeats interfere with gap repair to cause repeat expansions and chromosome breaks
Source: Nat Commun. 2023 Apr 29;14:2469. doi: 10.1038/s41467-023-37901-2 (PMC10148874; doi:10.1038/s41467-023-37901-2)
Supplement: Supplementary file 1 — Supplementary Information [file 41467_2023_37901_MOESM1_ESM.pdf]

Structure-forming CAG/CTG repeats interfere with gap repair to cause repeat expansions and chromosome breaks

Polleys. EJ et. al (2023)

Table of contents:

Supplemental methods
Supplementary table 1
Supplementary figures 1-6 and legends

Supplemental Methods:

Rad53 Phosphorylation - Time course collection was done at the same time as cells were collected for the kinetic Southern blot. Positive control cells were grown to log phase, treated with 0.035% MMS for 90 minutes. For each timepoint, 9.25×10^7 cells were collected. Protein was extracted via TCA method as previously described (DOI: 10.1128/mcb.14.2.923933.1994). Westerns were probed with anti-Rad53 antibody (1:1000; mouse monoclonal EL7.E1 Abcam Cat#AB166859).

Microcolony formation – G1 phase cells were micromanipulated onto solid YPGal media. Cells were observed 8 and 24 hours post manipulation and phenotypically scored. Phenotypic scoring is listed in source data.

Supplementary Table 1: Strains, Plasmids, Primers used

**Parent strain**		
YMV80	MATΔ::hisG hmlΔ::ADE1 hmrΔ::ADE1 ade1 lys5 ura3-52 trp1Δ HO ade3::GAL-HO leu2::cs	Vaze et al, 1999
no repeat	same as YMV80, but ilv6Δ::HPH	this study
**scrm$(CTG)_{70}$ template strains**		
CFY4765, 4766	same as YMV80, but ilv6Δ::(scrmCTG)70-HPH	this study
CFY5529, 5530	same as parent 4797; rad9::trp1	this study
CFY4946, 4947	same as parent 4797; rad51::kanMX	this study
CFY5689, 5690	same as parent 4797; rad9::trp1 rad51::kanMX	this study
**$(CAG)_{70}$ template strains**		
CFY4017,4018,4019,4020	same as YMV80, but ilv6Δ::(CAG)70-HPH	this study
**$(CTG)_{70}$ template strains**		
CFY4797, 4798	same as YMV80, but ilv6Δ::(CTG)70-HPH	this study
CFY5351, 5352	same as parent 4797; rad9::trp1	this study
CFY4948, 4949, 4950	same as parent 4797; rad51::kanMX	this study
CFY5691	same as parent 4797; rad9::trp1 rad51::kanMX	this study
CFY4879	same as parent 4797; mus81::kanMX	this study
CFY4856	same as parent 4797; mlh1::kanMX	this study
**Plasmids**		
pCF390	$(CTG)_{70}$-HPH	Su et al, 2015
pCF590	$(CAG)_{70}$-HPH	this study
pCF722, 723	scrm$(CTG)_{70}$-HPH	this study
pCF187	pRS414	
pCF582, 583	pRS414+NFS1	this study
**Primers**		
P1 - for scrm (CTG)70 and (CTG)70 templates forward	5' CCC AGG CCT CCA GTT TGC 3'	
P2 - for scrm (CTG)70 and (CTG)70 templates reverse	5’ TAA TAC GAC TCA CTA TAG GG 3’	
P1 - for (CAG)70 templates forward	5' CCG CCA GCT GAA GCT TGA AT 3'	
P2 - for (CAG)70 templates reverse	5' CAG TTT GCC CAT CCA CGT CA 3'	
P3 - 60 bp after the repeat for	5' TAC AAG GAC CCT TCG AGC CC 3'	
P4 - 60 bp after the repeat rev	5' GGG GAC GAG GCA AGC TAA AC 3'	
P5 - HPH locus forward	5' AAA TAG CTG CGC CGA TGG TTT C 3'	
P5 - HPH locus forward (ChIP only)	5' GAT TCC GGA AGT GCT TGA C 3'	
P6 - HPH locus reverse	5' CAG CGA TCG CAT CCA TGG CCT C 3'	
P7 - BUD3 locus forward	5’ ACT GCT GAA TTT CCG GTG GA 3’	
P8 - BUD3 locus reverse	5’ TCT GAG CAT TTC CTA ACA CGG T 3’	
P9 - LDB16 locus forward	5' CCA AAA CTC ATT CGT CAC CA 3'	
P10 - LDB16 locus reverse	5' ACA TGG GTT ACT GGC AGA AA 3'	
P11 - RNQ1 locus forward	5' GCT TTG GCG TCT TTG GCT TC 3'	
P12 - RNQ1 locus reverse	5' CTC CAA AGG AGG AAC CAC CG 3'	
ACT1 forward	5' TCC AGA TGG TCA AGT CAT CA 3'	
ACT1 reverse	5' TCG GCA ATA CCT GGG AAC AT 3'	

Supplementary Figure 1

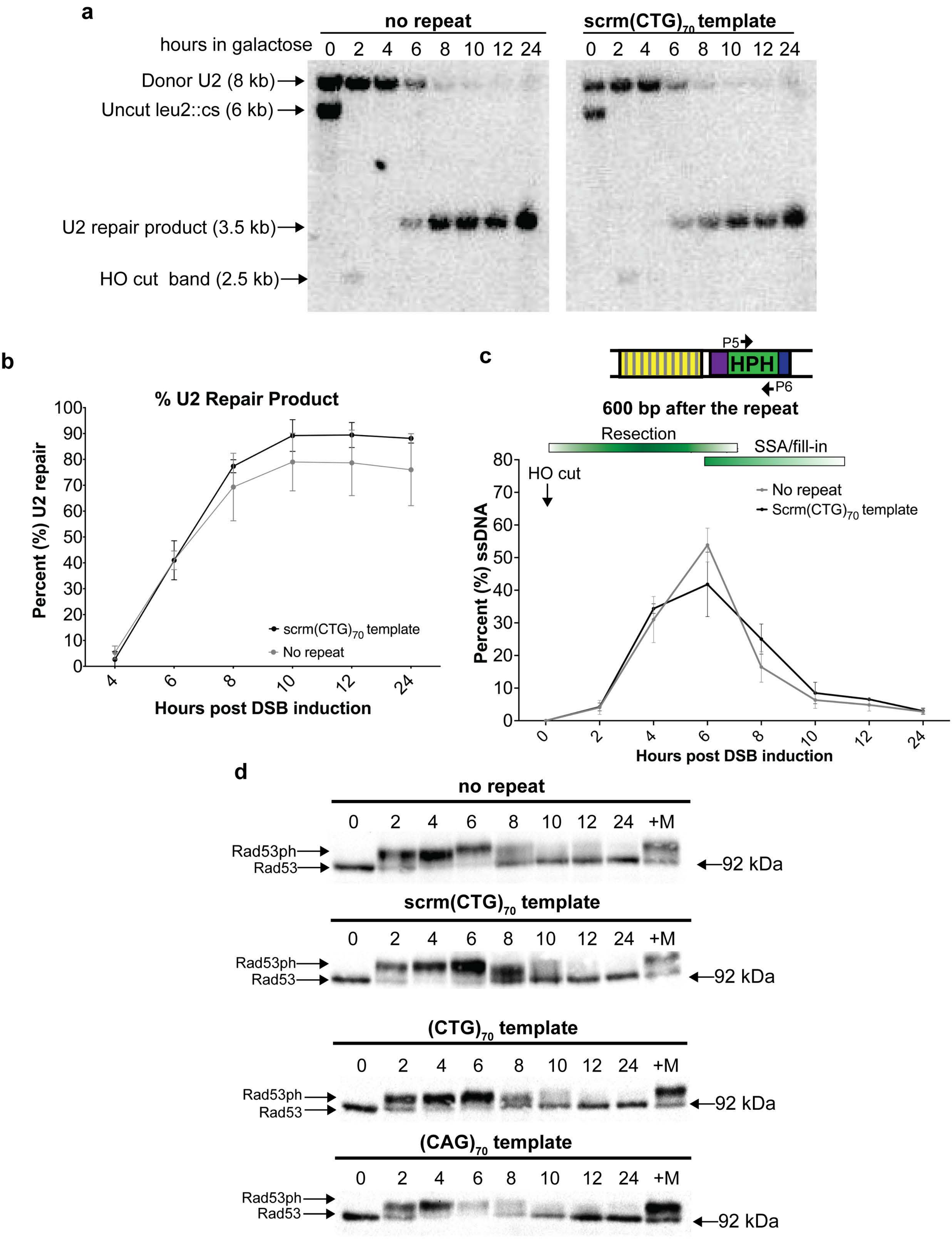

Supplementary Figure 1: The scrm(CTG)$_{70}$ strain has similar repair kinetics to the no repeat control. a) Southern blot analysis of no repeat (n=3) and scrm(CTG)70 (n=3) after addition of 2% galactose to induce a DSB within *LEU2*. Each n represents a biologically independent time course. Images are from the same Southern, scrm(CTG)70 is a different blot from the one in Figure 2a. **b)** U2 repair measurement (%) on Southern blots after DSB induction. Number of replicates measured: scrm(CTG)$_{70}$ n=3, no repeat=3 where each n represents biologically independent time courses. Graph shows mean ± SD. Individual experimental values can be found in source data. **c)** Formation and disappearance of ssDNA after the repeat locus post DSB induction, using primers P3 and P4 in the no repeat (n=4) and scrm(CTG)$_{70}$ (n=3) strains where each n represents biologically independent time courses. Graph shows mean ± SD; individual experimental values can be found in source data. **d)** Protein lysates of no repeat (n=3), scrm(CTG)$_{70}$ (n=3), (CTG)$_{70}$ (n=3) and (CAG)$_{70}$ (n=3) repeat strains were analyzed by Western blot for hyper-phosphorylated Rad53 at indicated timepoints post DSB induction. Each n value represents independent time courses. A positive control. (+M) of the no tract strain treated with 0.035% MMS for 90m was included on all blots. For **a-d)** source data are provided as a Source Data file.

Supplementary Figure 2

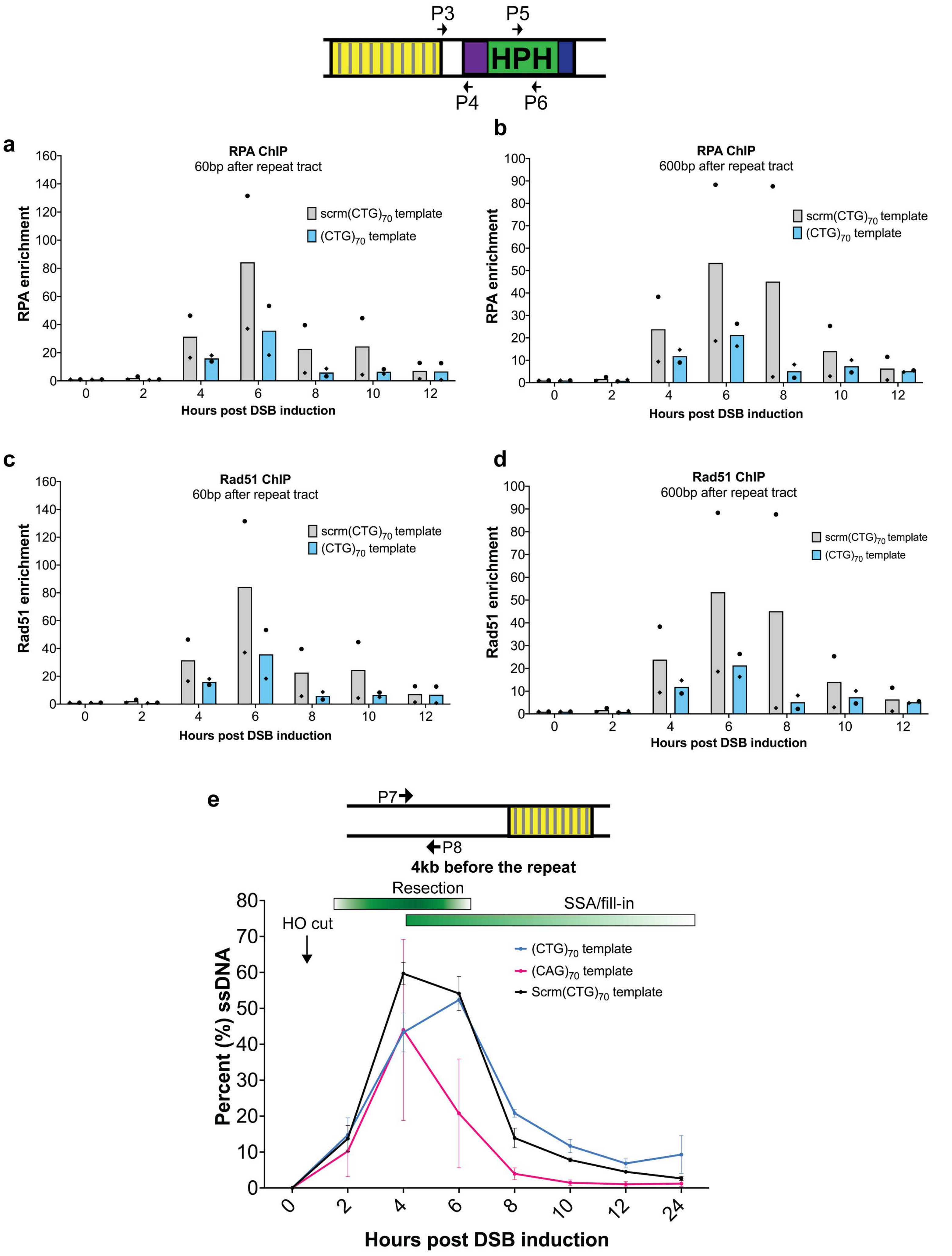

Supplementary Figure 2: Enrichment of RPA and Rad51 on scrm$(CTG)_{70}$ and $(CTG)_{70}$ templates. a) Enrichment of RPA 60 bp (P3 & P4 amplicon) and **b)** 600 bp (P5 & P6 amplicon) after the repeat in scrm$(CTG)_{70}$ (n=2) and $(CTG)_{70}$ (n=2) template strains following DSB induction. **c)** Enrichment of Rad51 60 bp (P3 & P4 amplicon) and **d)** 600 bp (P5 & P6 amplicon) after the repeat in scrm$(CTG)_{70}$ (n=2) and $(CTG)_{70}$ (n=2) template strains following DSB induction. For all ChIP graphs in a-d, the bars depict the mean; • and ♦ indicate one experimental replicate. **e)** ssDNA abundance in a region 4 kb before the repeat locus with primers P7 and P8. Number of replicates: scrm$(CTG)_{70}$ n=3, $(CTG)_{70}$ n=3 and $(CAG)_{70}$ n=2 where each n represents biologically independent time courses. Graph shows mean ± SD. For **a-e)** source data are provided as a Source Data file.

Supplementary Figure 3

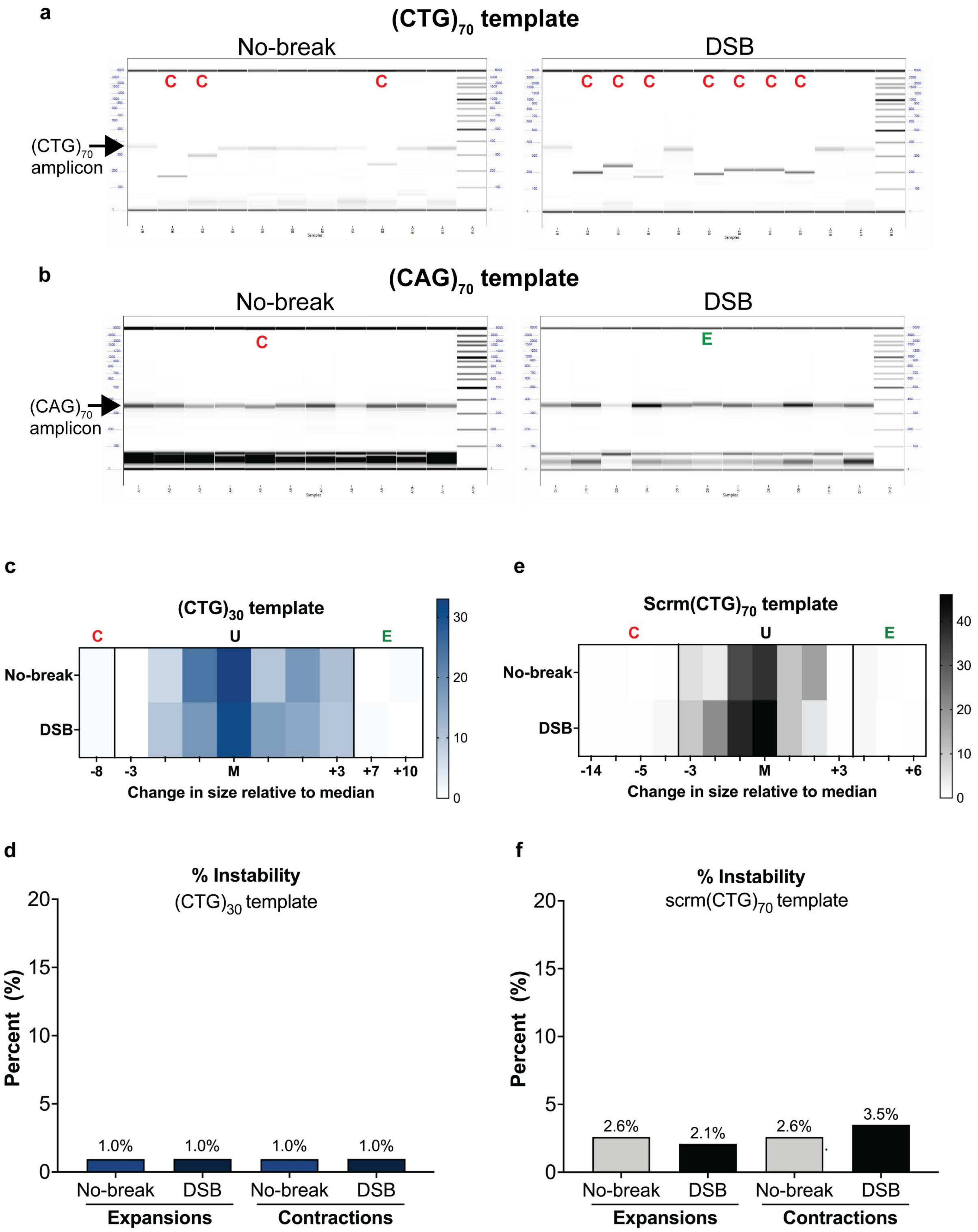

Supplementary Figure 3

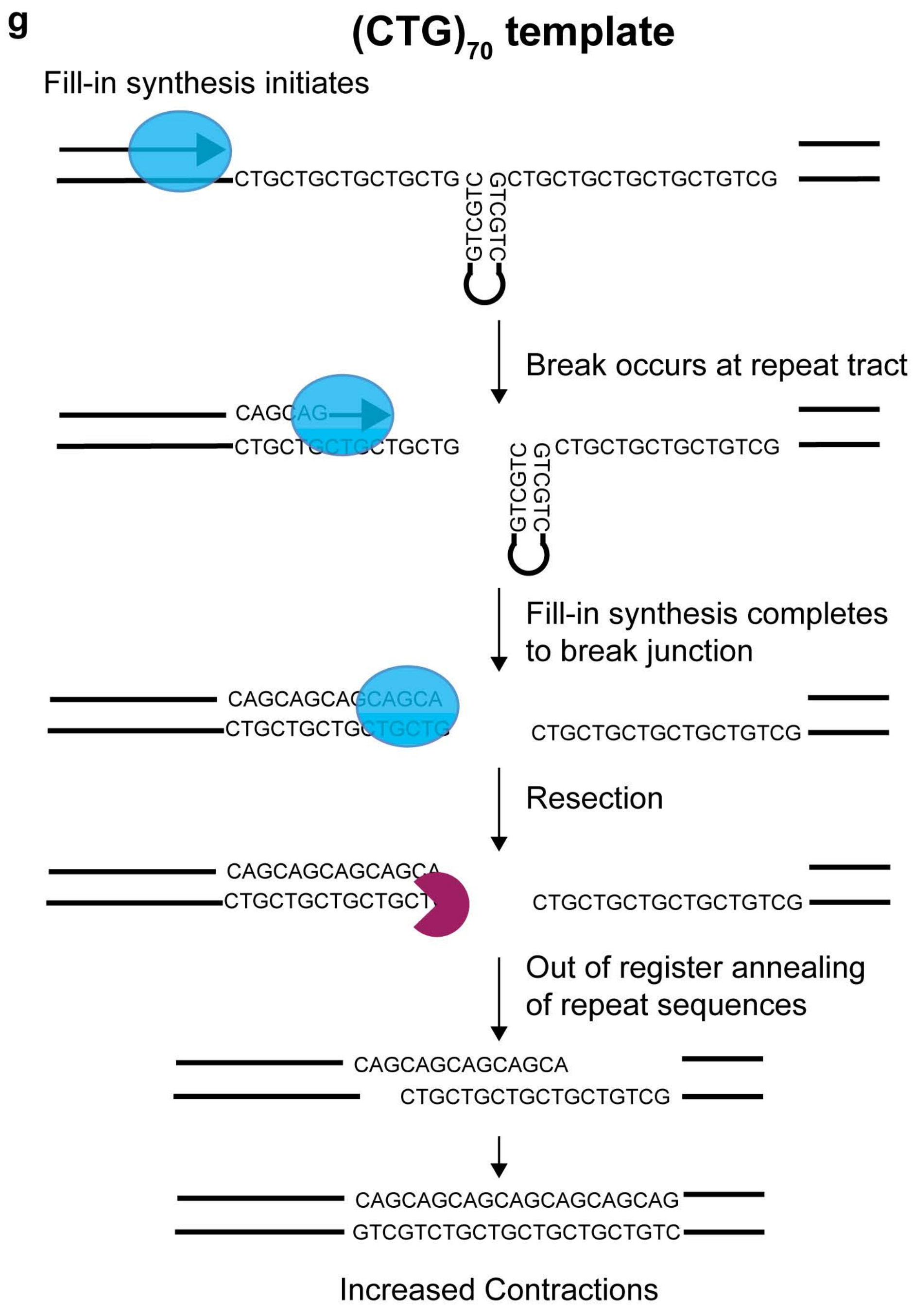

Supplementary Figure 3: Visualization of $(CAG)_{70}$ and $(CTG)_{70}$ sizes and quantification of scrm$(CTG)_{70}$ and $(CTG)_{30}$ instability. a) Capillary gel electrophoresis images of $(CTG)_{70}$ repeat tract amplification in both the no-break and DSB conditions. Arrow points to unchanged size, C represents contracted samples. **b)** Capillary gel electrophoresis images of $(CAG)_{70}$ repeat tract amplification in both the no-break and DSB conditions. Arrow points to unchanged size, C represents contracted samples while E represents expanded samples. **c)** Heatmap of amplicon sizes for the $(CTG)_{30}$ template strain. Abbreviations are the same as in figure 3a. Total number of PCR reactions represented: no break n=105, break n=103. **d)** Quantification of expansions and contractions of the (CTG)30 sequence determined from fig. c). **e)** Heat map of amplicon sizes for the scrm$(CTG)_{70}$ sequence. Abbreviations are the same as Figure 3a. Total number of PCR reactions represented: no-break n=115, break n=141. **f)** Quantification of expansions and contractions of the scrm$(CTG)_{70}$ sequence determined from fig. e). **g)** Model for repeat contractions due to ssDNA breaks at the $(CTG)_{70}$ template. Resection and annealing of the U2 sequences occur unimpeded in the $(CTG)_{70}$ template strains. The ssDNA is left unprotected such that CTG hairpins can form and are targets of nucleolytic cleavage. Polymerase fill-in occurs independent of the break. Once the filled in strand is double stranded, resection and out of register alignment at the repeat tract occurs resulting in contractions. For **c-f)** source data are provided as a Source Data file.

Supplementary Figure 4

a

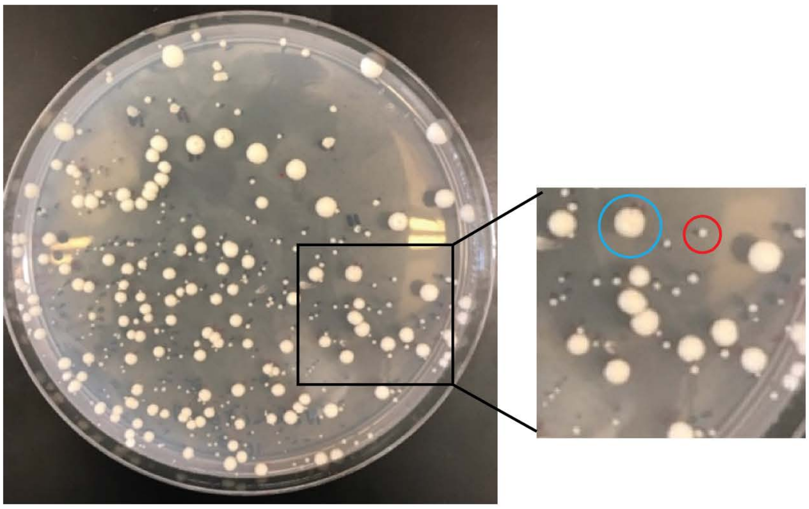

b

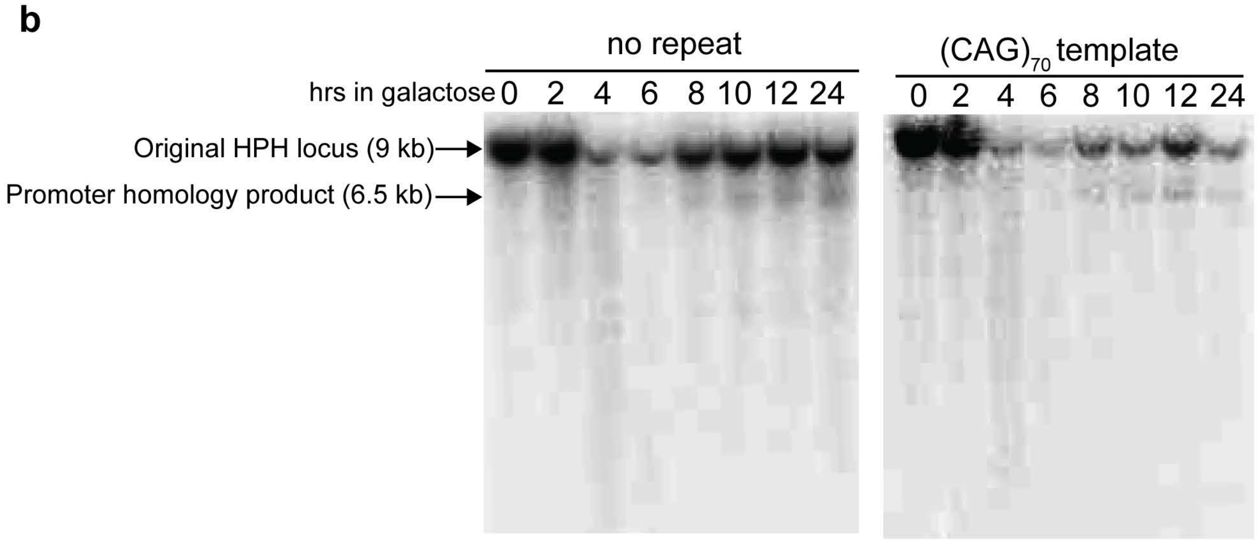

c

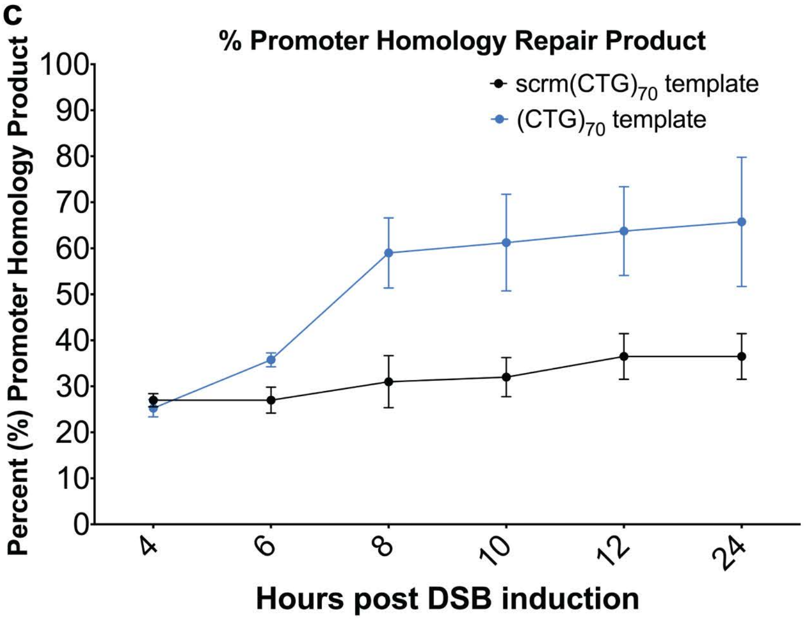

d

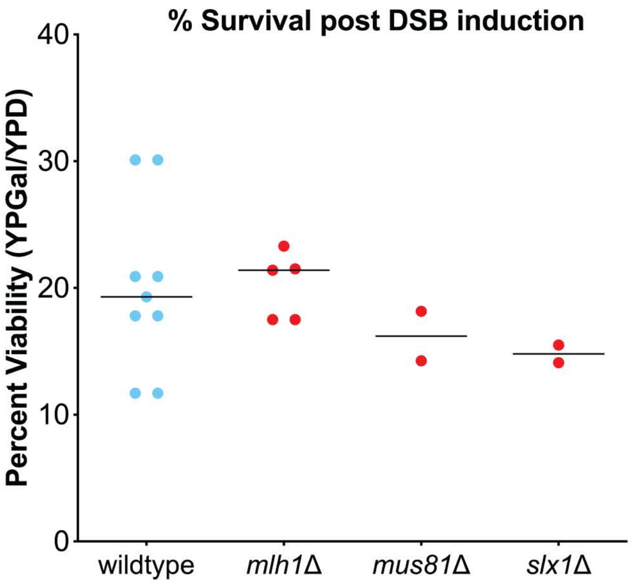

Supplementary Figure 4

e

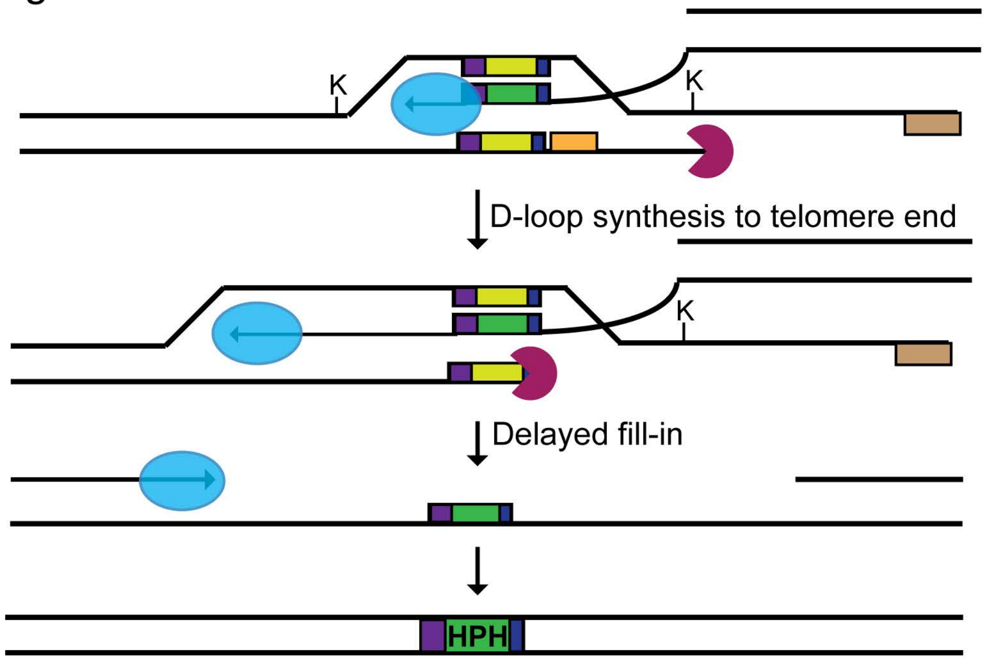

f

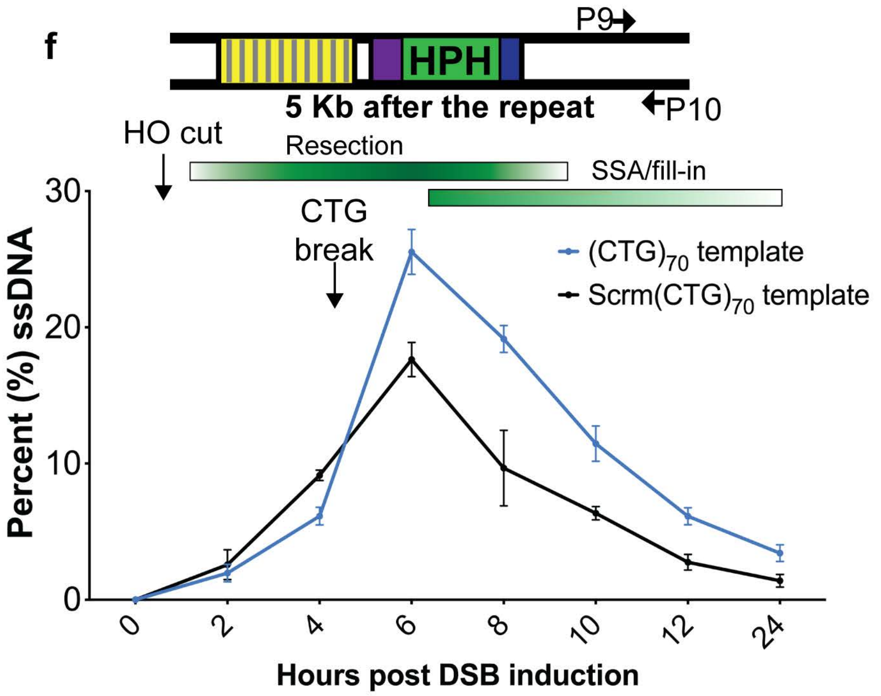

g

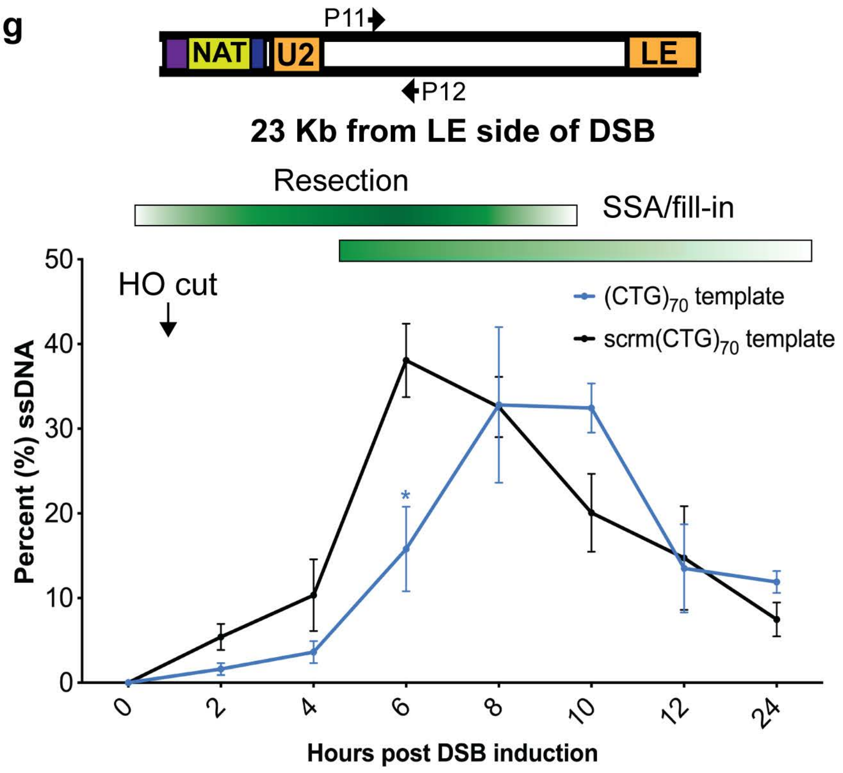

Supplementary Figure 4: *NFS1* complemented colonies and resection on the other side of the DSB in the $(CTG)_{70}$ template strain. a) There are two populations of resultant colonies from the $(CTG)_{70}$ strain containing the *NFS1* plasmid plated on break induction media. Colony circled in blue is a large colony, colony circled in red is a small colony. **b)** No tract and $(CAG)_{70}$ template Southern blots of KpnI digested DNA probed with a fragment to the *HPH* locus. Representative Southern shown; number of replicates: No repeat (n=2) and $(CAG)_{70}$ (n=2). **c)** Promoter homology repair measurement (%) on Southern blots after DSB induction. Number of replicates measured: scrm$(CTG)_{70}$ n=2, $(CTG)_{70}$ n=4. Graph shows mean ± SD. **d)** Percent viability of *mlh1*Δ (n=5), *mus81* Δ (n=2) and *slx1*Δ (n=2) compared to wildtype $(CTG)_{70}$ template strain (n=9) where n represents assays from biologically independent experiments. Statistics comparing *mlh1*Δ to wildtype were determined using an unpaired, two-tailed Student's t-test (p=0.95). **e)** Model for D-loop formation and filling in of BIR repair events in colonies with a $(CTG)_{70}$ ssDNA template that has broken (see Fig. 4a). BIR initiates from TEF promoter homology and D-loop synthesis occurs to the telomere end. Exonucleolytic degradation of the template is delayed as D-loop formation at the site of the TEF promoter occurs (see P11-12 amplicon data, Figure S4d). After leading strand BIR synthesis, filling in of the opposite strand is delayed in time (see P9-P10 amplicon data, Fig 4e). **f)** Resection and gap filling kinetics of a region 5 kb after the repeat was determined for scrm$(CTG)_{70}$ (n=2) and $(CTG)_{70}$ (n=4) strains. Graph shows mean ± SD. **g)** Resection and fill-in kinetics of strains with a $(CTG)_{70}$ repeat template is impaired on the other (U2) side of the DSB. Percent ssDNA from DSB induced strains 15 kb to the left of the DSB. Graph shows mean ± SD. Number of replicates: scrm$(CTG)_{70}$ (n=3), $(CTG)_{70}$ (n=3), where n is independent time courses; statistical significance determined using an unpaired Student's t-test using a two-stage step-up with a false-discovery rate of 1% (Benjamini, Krieger, and Yekutieli). For **b,c,d,f,** g) source data are provided as a Source Data file.

Supplementary Figure 5

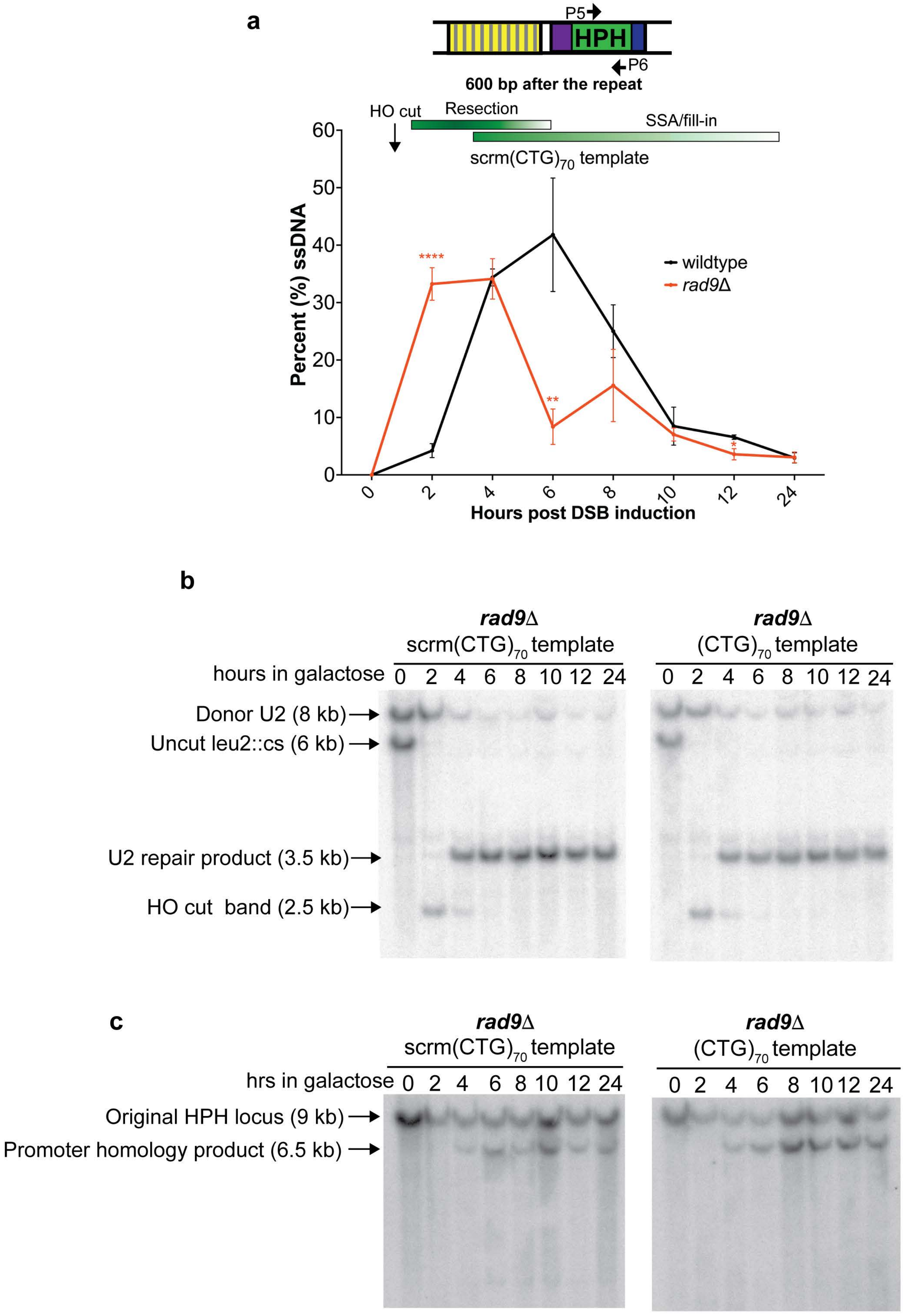

Supplementary Figure 5

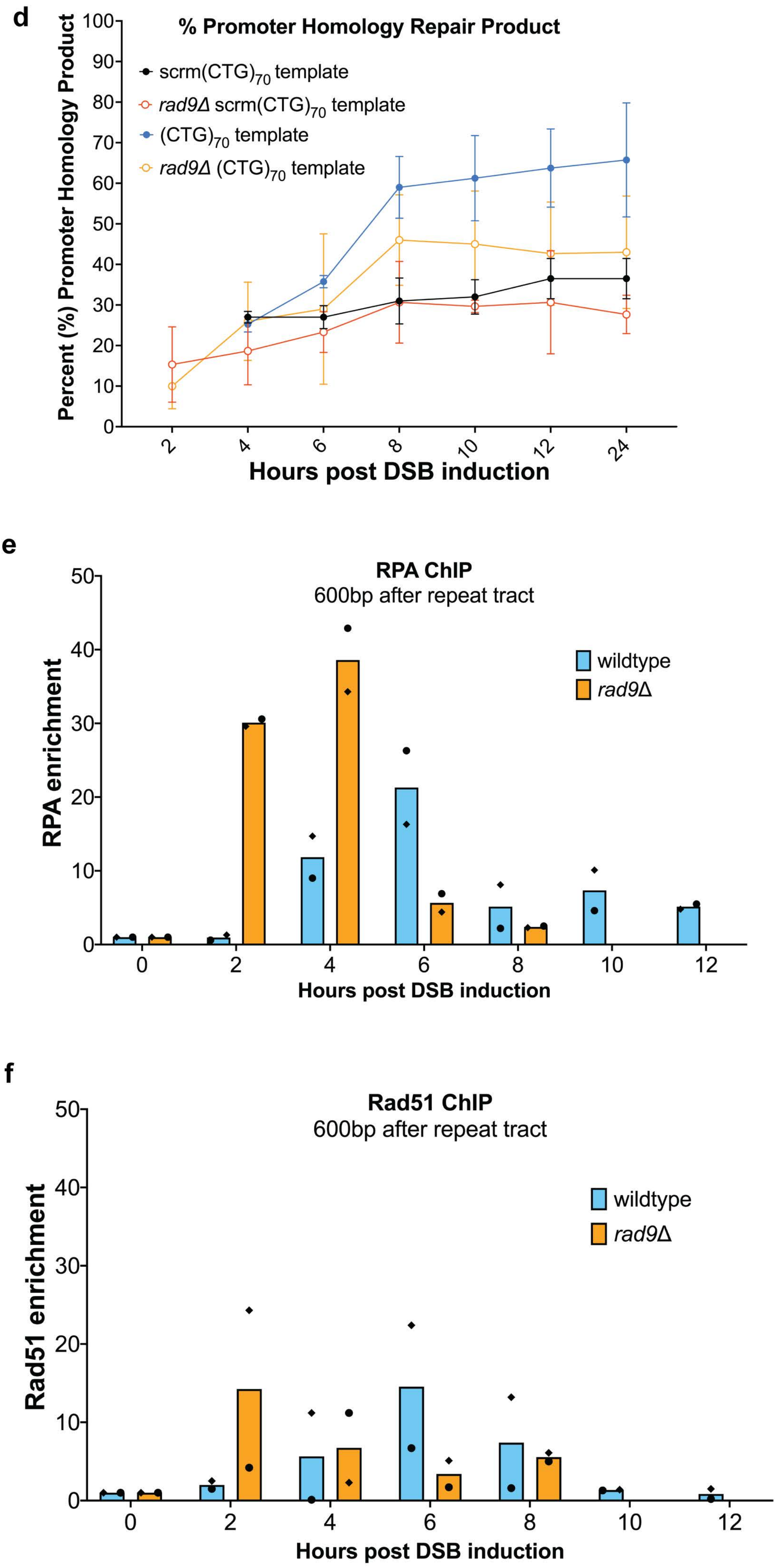

Supplementary Figure 5: Deletion of Rad9 speeds up resection and repair. a) Resection and fill-in kinetics of the scrm$(CTG)_{70}$ template construct in wildtype and *rad9*Δ strains. Percent ssDNA after the repeat locus from DSB induced strains was determined as in Figure 2b. Number of replicates: scrm$(CTG)_{70}$ (n=3), *rad9*Δ scrm$(CTG)_{70}$ (n=3) where each n represents biologically independent time courses. Statistical analysis done using an unpaired Student's t-test using a two-stage step-up with a false-discovery rate of 1% (Benjamini, Krieger, and Yekutieli); all p-values listed in source data. **b)** Southern blot analysis after addition of 2% galactose to induce a DSB within *LEU2* of the scrm$(CTG)_{70}$ and $(CTG)_{70}$ template constructs in *rad9Δ* mutants. Representative Southern shown; number of replicates: *rad9Δ* scrm$(CTG)_{70}$ (n=3) and *rad9*Δ $(CTG)_{70}$ (n=3) where each n represents biologically independent time courses. **c)** Kinetic Southern blots were stripped and probed with a fragment to the *HPH* locus. Representative Southern shown; number of replicates: scrm$(CTG)_{70}$ (n=3) and $(CTG)_{70}$ (n=3) each n represents biologically independent time courses. **d)** Promoter homology repair measurement (%) on Southern blots after DSB induction. Number of replicates measured: scrm$(CTG)_{70}$ template strain (n=2), *rad9*Δ in scrm$(CTG)_{70}$ template strain (n=3), $(CTG)_{70}$ template strain (n=4), *rad9*Δ in $(CTG)_{70}$ template strain (n=3). Each n represents biologically independent time courses. Graph shows mean ± SD. All measurement values and statistical analysis (p-values) can be found in source data. Enrichment of **e)** RPA and **f)** Rad51 occurs earlier 600 bp after $(CTG)_{70}$ repeat tract in the *rad9*Δ mutant following DSB induction compared to wildtype. Independent biological replicates for wildtype (n=2) and *rad9*Δ (n=2) where each n represents independent time courses. Enrichment adjacent to the $(CTG)_{70}$ repeat was determined using primers P3 & P4 and calculated using absolute quantity and normalized to *ACT1*. Bars on graph depict mean; • and ♦ indicate one experimental replicate. For **a-f)** source data are provided as a Source Data file.

Supplementary Figure 6

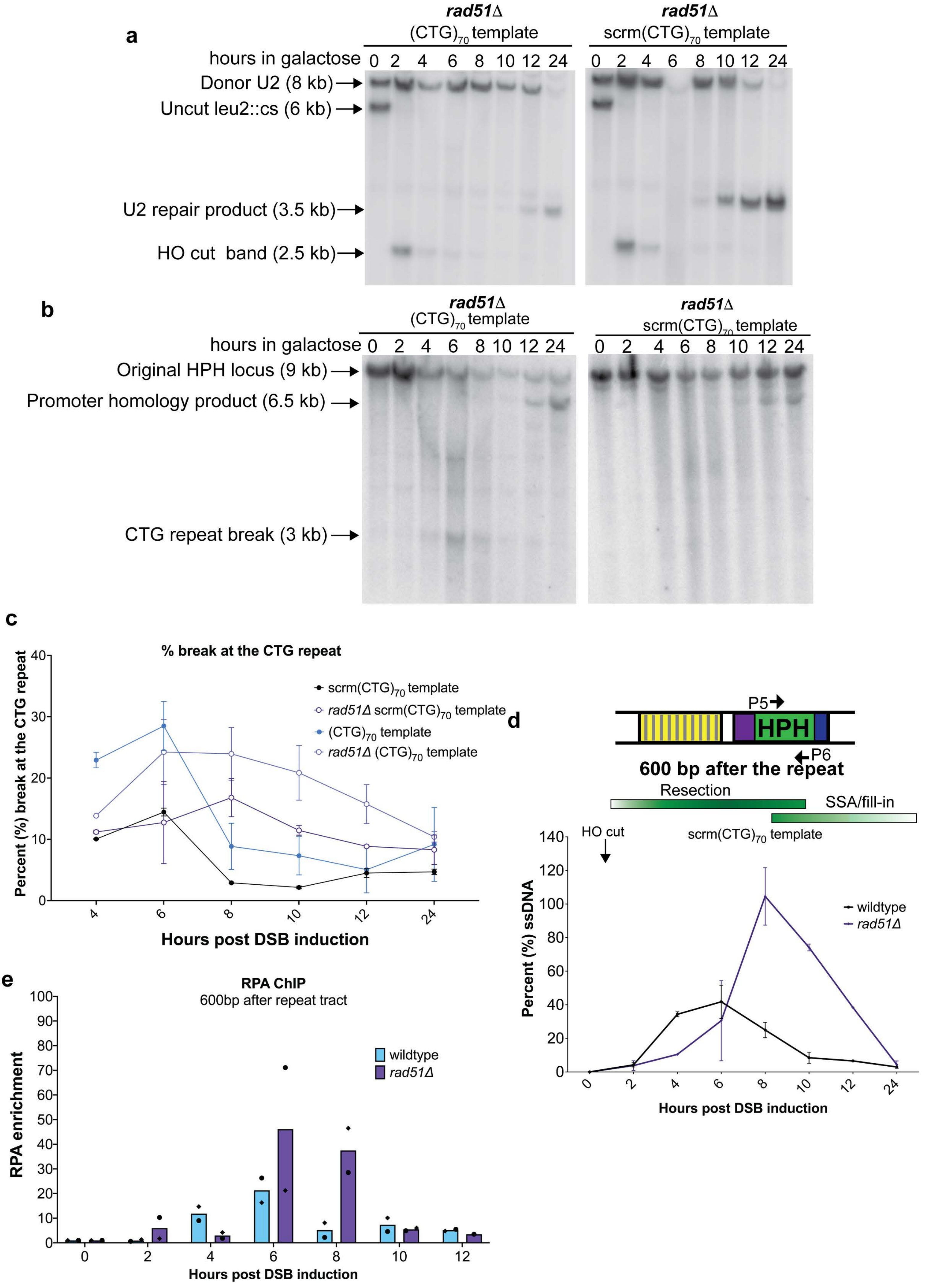

Supplementary Figure 6

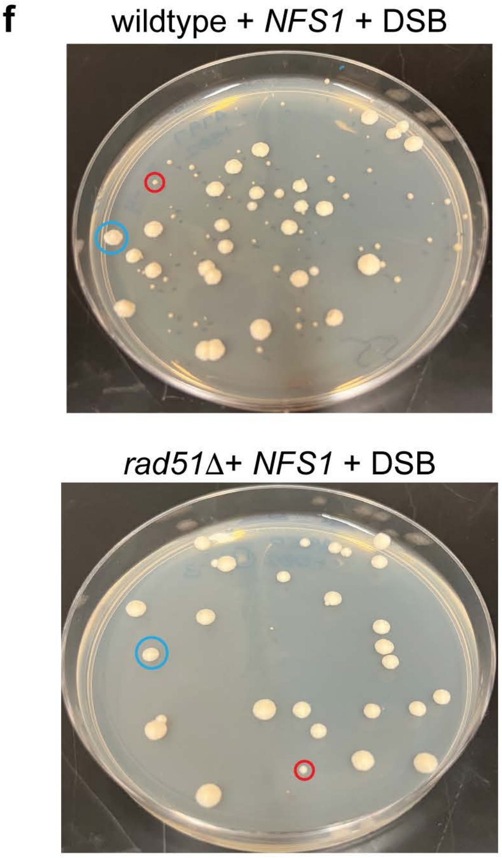

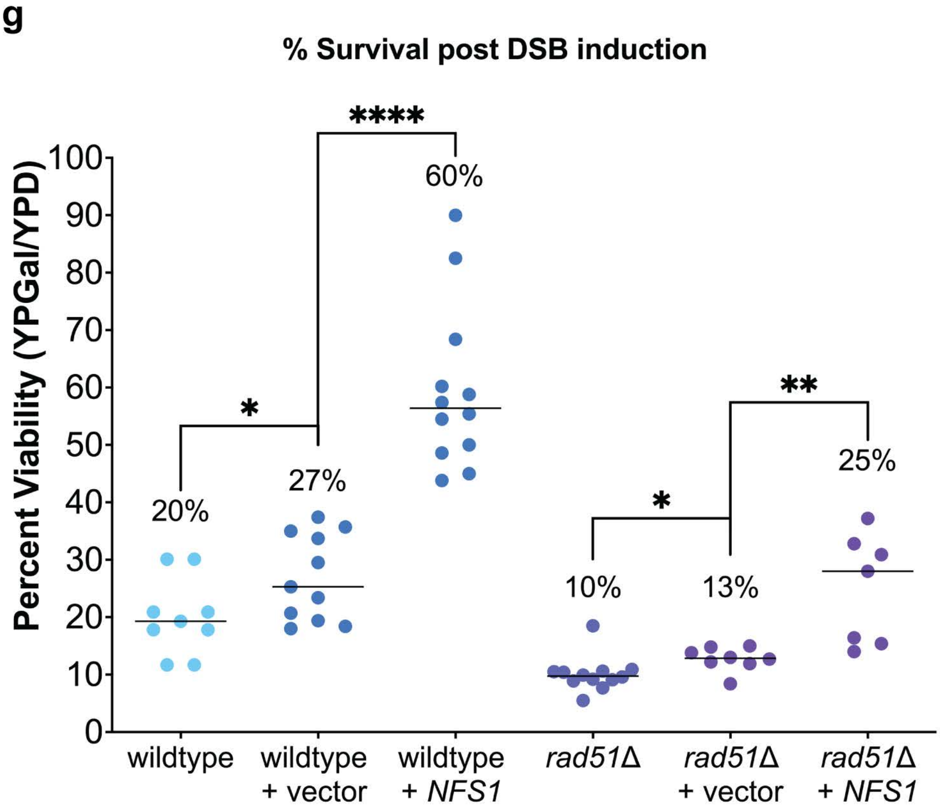

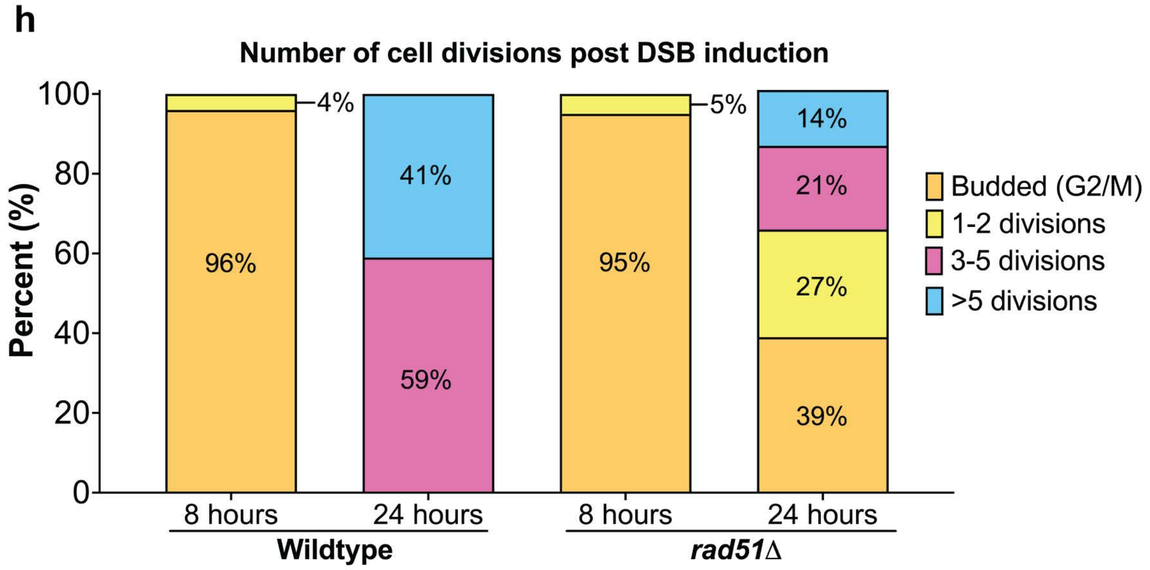

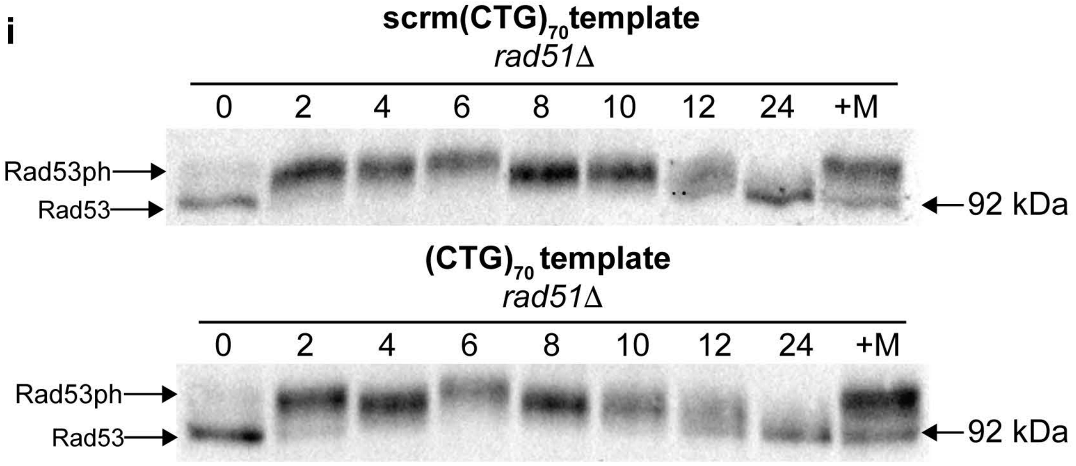

Supplementary Figure 6: Deletion of Rad51 delays BIR repair and resection through the repeat tract. a) Southern blot of U2 repair for the scrm$(CTG)_{70}$ (n=2) and $(CTG)_{70}$ (n=2) template strains in *rad51*Δ mutants. Representative Southern shown; each n represents biologically independent time courses. Probe is to a portion of the *LEU2* gene (probe location noted in Figure 1a). **b)** Promoter homology repair measurement (%) on post DSB induction of *rad51*Δ mutants in the scrm$(CTG)_{70}$ (n=2) and $(CTG)_{70}$ (n=2) strains were probed with a fragment to the *HPH* locus 600 bp downstream of the repeat tract. Representative Southern shown; each n represents biologically independent time courses. **c)** CTG repeat breakage (%) on Southern blots after DSB induction. Graph shows mean ± SD. Number of replicates: scrm$(CTG)_{70}$ (n=3) , *rad51*Δ scrm $(CTG)_{70}$ (n=2), $(CTG)_{70}$ (n=4), and *rad51*Δ $(CTG)_{70}$ (n=2) where each n represents biologically independent time courses. Graph shows mean ± SD; all experimental values listed in source data. **d)** Resection and fill-in kinetics of the scrm$(CTG)_{70}$ template construct in wildtype (n=3) and *rad51*Δ (n=2) strains where each n represents biologically independent time courses. Percent ssDNA 600 bp after the repeat locus after DSB induction. Graph shows mean ± SD. **e)** Enrichment of RPA 600 bp after the $(CTG)_{70}$ repeat tract in wildtype and *rad51*Δ mutant following DSB induction. Independent biological replicates for wildtype (n=2) and *rad51*Δ (n=2) where each n represents independent time courses. Enrichment adjacent to the $(CTG)_{70}$ repeat was determined using P5 & P6 and calculated using absolute quantity and normalized to *ACT1*. Bars on graph depicts mean; • and ♦ indicate one experimental replicate. **f)** There are two populations of resultant colonies from the $(CTG)_{70}$ strain containing the *NFS1* plasmid plated on break induction media. Small colonies are less observed in *rad51Δ* $(CTG)_{70}$ strains complemented with NFS1. Colony circled in blue is a large colony, colony circled in red is a small colony. **g)** Percent viability of $(CTG)_{70}$ template (n=9), $(CTG)_{70}$ template + vector (n=11) and $(CTG)_{70}$ template+*NFS1* (n=12) are shown. Student's t-test (two-tailed, unpaired) statistical comparison of $(CTG)_{70}$ no vector to $(CTG)_{70}$+vector is p=0.04. Student's t-test (two-tailed, unpaired) statistical comparison of $(CTG)_{70}$+vector to $(CTG)_{70}$+*NFS1* is p<0.0001. Percent viability of *rad51Δ* $(CTG)_{70}$ (n=12), *rad51Δ* $(CTG)_{70}$ + vector (n=8), *rad51Δ* $(CTG)_{70}$ + *NFS1* (n=7). Student's t-test (two-tailed, unpaired) statistical comparison of *rad51Δ* $(CTG)_{70}$ no vector to *rad51Δ* $(CTG)_{70}$+vector is p=0.04. Student's t-test (two-tailed, unpaired) statistical comparison of $(CTG)_{70}$+*NFS1* is p= 0.003. Each n value represents assays from biologically independent experiments. **h)** Recovery at 8 and 24 hours post-DSB formation of wildtype (n=92) and *rad51*Δ mutant (n=135) cells for the $(CTG)_{70}$ template strain. Each n represents a single cell that was allowed to divide over time after micromanipulation onto DSB induction media. **i)** Protein lysates of *rad51Δ* scrm$(CTG)_{70}$ (n=2) and *rad51Δ* $(CTG)_{70}$ (n=2) repeat strains were analyzed by Western blot for hyper-phosphorylated Rad53 at indicated timepoints post DSB induction. Each n value represents independent time courses. A positive control (+M) of the no tract strain treated with 0.035% MMS for 90m was included on all blots. For **a-i)** source data are provided as a Source Data file.
