# Supplementary material for: Structure-forming CAG/CTG repeats interfere with gap repair to cause repeat expansions and chromosome breaks
Source: Nat Commun. 2023 Apr 29;14:2469. doi: 10.1038/s41467-023-37901-2 (PMC10148874; doi:10.1038/s41467-023-37901-2)
Supplement: Supplementary file 3 — Reporting Summary [file 41467_2023_37901_MOESM3_ESM.pdf]

nature portfolio

Corresponding author(s): Catherine Freudenreich

Last updated by author(s): March 17th 2023

Reporting Summary

Nature Portfolio wishes to improve the reproducibility of the work that we publish. This form provides structure for consistency and transparency in reporting. For further information on Nature Portfolio policies, see our Editorial Policies and the Editorial Policy Checklist.

Statistics

For all statistical analyses, confirm that the following items are present in the figure legend, table legend, main text, or Methods section.

n/a	Confirmed	
☐	☒	The exact sample size (*n*) for each experimental group/condition, given as a discrete number and unit of measurement
☐	☒	A statement on whether measurements were taken from distinct samples or whether the same sample was measured repeatedly
☐	☒	The statistical test(s) used AND whether they are one- or two-sided<br>*Only common tests should be described solely by name; describe more complex techniques in the Methods section.*
☒	☐	A description of all covariates tested
☒	☐	A description of any assumptions or corrections, such as tests of normality and adjustment for multiple comparisons
☐	☒	A full description of the statistical parameters including central tendency (e.g. means) or other basic estimates (e.g. regression coefficient) AND variation (e.g. standard deviation) or associated estimates of uncertainty (e.g. confidence intervals)
☐	☒	For null hypothesis testing, the test statistic (e.g. *F*, *t*, *r*) with confidence intervals, effect sizes, degrees of freedom and *P* value noted<br>*Give P values as exact values whenever suitable.*
☒	☐	For Bayesian analysis, information on the choice of priors and Markov chain Monte Carlo settings
☒	☐	For hierarchical and complex designs, identification of the appropriate level for tests and full reporting of outcomes
☒	☐	Estimates of effect sizes (e.g. Cohen's *d*, Pearson's *r*), indicating how they were calculated

Our web collection on statistics for biologists contains articles on many of the points above.

Software and code

Policy information about availability of computer code

Data collection	Agilent Fragment Analyzer V. 1.0.2.9, Applied Biosystems QuantStudio 6 v1.3
Data analysis	Biorad Image Lab v. 6.1.0; GE Imagequant TL v8.2.0, GraphPad Prism v 9.3.1, Microsoft Excel v. 16.59, Agilent Prosize 2.0 v. 1.3.1.1; ImageJ v. 1.53

For manuscripts utilizing custom algorithms or software that are central to the research but not yet described in published literature, software must be made available to editors and reviewers. We strongly encourage code deposition in a community repository (e.g. GitHub). See the Nature Portfolio guidelines for submitting code & software for further information.

Data

Policy information about availability of data

All manuscripts must include a data availability statement. This statement should provide the following information, where applicable:

- Accession codes, unique identifiers, or web links for publicly available datasets
- A description of any restrictions on data availability
- For clinical datasets or third party data, please ensure that the statement adheres to our policy

Source data are provided with this paper. The authors declare that data supporting the findings in this study are available within the paper and its supplementary information files. Southern and western blot images detailed in fig. 2a, fig. 4d, and supplemental figures 1a, 1d, 4b, 5b, 5c, 6a, 6b, and 6i are representative blots

from several replicate experiments (n=2 or more). All Southern blots were quantified, and values are available in the source data. Replicate blots are not shown, but available on request.

Human research participants

Policy information about studies involving human research participants and Sex and Gender in Research.

Reporting on sex and gender	N/A
Population characteristics	N/A
Recruitment	N/A
Ethics oversight	N/A

Note that full information on the approval of the study protocol must also be provided in the manuscript.

Field-specific reporting

Please select the one below that is the best fit for your research. If you are not sure, read the appropriate sections before making your selection.

☒ Life sciences ☐ Behavioural & social sciences ☐ Ecological, evolutionary & environmental sciences

For a reference copy of the document with all sections, see nature.com/documents/nr-reporting-summary-flat.pdf

Life sciences study design

All studies must disclose on these points even when the disclosure is negative.

Sample size	For repair Southerns, resection assays and viability assays, sample sizes were determined using previously published literature (doi: 10.1016/s1097-2765(02)00593-2). Sample sizes for instability analysis was determined using previously published literature using similar assay systems (https://doil.org/10.7554/eLife.53362). Sample size was determined by the number needed to determine statistical significance balanced by the practicality of the number of PCR reactions that could be done with available resources (as stated in https://doil.org/10.7554/eLife.53362).
Data exclusions	One (CAG)70 template strain repair Southern was not quantified due to poor image quality. The data in the paper represent an n=3 for visual representation and resection assays but quantified repair measurement is an n=2. For Figure S6h, colonies that were pinned onto solid media and did not bud at 8 hours post-DSB induction were treated as dead cells and excluded from analysis.
Replication	All kinetic Southerns, resection assays and chromatin immunoprecipitation experiments were performed at least 2 times. Viability assays were performed at least 2 times. Instability frequencies were determined from a n>100 independent colonies from each experimental condition (no break and DSB). When applicable statistical analysis confirmed reproducibility of the results. Fisher's exact test was used for determination of significance for changes in repeat instability. Student's t-test was used for determining significance for changes in viability. Paired Studen't t-tests were used for determining statistical significance for paired samples across time for the ChIP analysis, quantified repair Southerns and resection assays. Independent colonies of each strain were used in each replicate analysis. All attempts at experimental replication were successful.
Randomization	For instability assays, random colonies were picked from the plates for each condition (no break; DSB) to ensure we weren't biasing our data toward expansions or contractions of the repeat tract. For other molecular/biochemical assays randominzation is not relevant as they are done with bulk cultures and the whole population of cells is assayed at once. All strains regardless of genotype were treated equivalently.
Blinding	Blinding is not relevant to our studies as all mutant strains are being compared to wildtype.

Reporting for specific materials, systems and methods

We require information from authors about some types of materials, experimental systems and methods used in many studies. Here, indicate whether each material, system or method listed is relevant to your study. If you are not sure if a list item applies to your research, read the appropriate section before selecting a response.

Materials & experimental systems

n/a	Involved in the study
☐	☒ Antibodies
☒	☐ Eukaryotic cell lines
☒	☐ Palaeontology and archaeology
☒	☐ Animals and other organisms
☒	☐ Clinical data
☒	☐ Dual use research of concern

Methods

n/a	Involved in the study
☒	☐ ChIP-seq
☒	☐ Flow cytometry
☒	☐ MRI-based neuroimaging

Antibodies

Antibodies used	AS07 214 (Anti-RPA) by Agrisera; PA5-34905 (Anti-yRad51) by Thermo Fischer Scientific; AB166859 (EL7.E1; anti-yRad53) by Abcam
Validation	For the Rad51 ChIP, we used the same anti-Rad51 antibody as has been published for similar ChIP experiments (https://doi.org/10.1038/s41467-020-16997-w). For the RPA ChIP, we used the same anti-RPA antibody that has been used for similar ChIP experiments (https://doi.org/10.7554/eLife.21687). For the Rad53-ph antibody, we used the same antibody as shown in (http://doi.org/10.1016/j.molcel.2016.12.003) and was validated by Abcam for Western blotting.

March 2021
